# Melatonin downregulates angiogenesis and lymphangiogenesis by regulating tumor-associated macrophages via NLRP3 inflammasomes in lung adenocarcinoma

**DOI:** 10.18632/aging.206057

**Published:** 2024-09-03

**Authors:** Zhewei Zhao, Dongjie Ma, Yingzhi Qin, Yuan Xu, Shanqing Li, Hongsheng Liu

**Affiliations:** 1 Department of Thoracic Surgery, Peking Union Medical College Hospital, Chinese Academy of Medical Sciences and Peking Union Medical College, Beijing 100730, China

**Keywords:** lung adenocarcinoma, tumor microenvironment, angiogenesis and lymphangiogenesis, tumor-associated macrophages, melatonin

## Abstract

Tumor-associated macrophages (TAMs), present within the tumor microenvironment (TME), strictly modulate tumor angiogenesis and lymphangiogenesis. Nevertheless, the associated signaling networks and candidate drug targets for these events remains to be elucidated. Given its antioxidative activities, we speculated that melatonin may reduce pyroptosis, and thereby modulate both angiogenesis and lymphangiogenesis. We revealed that a co-culture of A549 cells and THP-1 macrophages strongly enhanced expressions of the NLRP3 inflammasome axis members, and augmented angiogenesis and lymphangiogenesis. Next, we overexpressed NLRP3 in the A549 cells, and demonstrated that excess NLRP3 expression substantially upregulated VEGF and CXCL cytokine expressions, and enhanced lymphatic endothelial cells (LECs) tube formation. In contrast, NLRP3 inhibition produced the opposite effect. In addition, relative to controls, melatonin administration strongly inhibited the NLRP3 inflammasome axis, as well as angiogenesis and lymphangiogenesis in the co-culture system. Subsequent animal experiments using a Lewis Lung Carcinoma (LLC) subcutaneous tumor model in mice corroborate these findings. Melatonin treatment and NLRP3 knockdown significantly inhibit tumor growth and downregulate NLRP3 and IL-1β expression in tumor tissues. Furthermore, melatonin downregulates the expression of angiogenic and lymphangiogenic markers in tumor tissues. Taken together, the evidence suggested that a THP-1 macrophage and A549 cell co-culture stimulates angiogenesis and lymphangiogenesis via the NLRP3 axis. Melatonin protected against the TAMs- and NLRP3 axis-associated promotion of the aforementioned events *in vitro* and *in vivo*. Hence, melatonin is a promising candidate for managing for tumor-related angiogenesis and lymphangiogenesis in lung adenocarcinoma.

## INTRODUCTION

Lung cancer (LC) has the largest incidence and mortality among all cancers worldwide, and it results in approximately 1.9 million deaths per year. About 85% of LC patients exhibit non-small cell LC (NSCLC), with adenocarcinoma as the most frequent subtypes, and 56% of patients have metastasis [1]. Angiogenesis and lymphangiogenesis are critical regulators of tumor development and metastasis, and these, in turn, are regulated by an intricate network of signaling pathways in the tumor microenvironment (TME) [2, 3].

TME constitutes a complex ecosystem harboring various cell types, extracellular matrix components, and signaling molecules [4]. It serves an essential function in tumor initiation, progression, and therapeutic response. Tumor-associated macrophages (TAMs) are key components of the TME, and their polarization state controls tumor angiogenesis [5]. For example, when polarized to a pro-tumoral phenotype, TAMs stimulate tumor growth and angiogenesis. Alternately, when polarized to an anti-tumoral phenotype, they suppress tumor development while upregulating immune activation [6]. A delicate balance between the two aforementioned phenotypes determines the overall nature of immune response in the TME.

Emerging evidence suggests that pyroptosis strongly regulates tumor progression [7, 8]. Moreover, the NLRP3 inflammasome is a potent modulator of pyroptotic cell death. The NLRP3 inflammasome is a cytosol-based complex of proteins responsible for innate immune response modulation [9]. Its stimulation enhances pro-inflammatory cytokine production, namely, interleukin IL-1β and IL-18. More recently, it was revealed that the NLRP3 inflammasome also regulates tumor progression [10, 11]. Several investigations reported that NLRP3 expression in TAMs was strongly correlated with tumor progression and metastasis [12, 13]. Nevertheless, the complex signaling networks associated with the NLRP3-mediated regulation of tumor angiogenesis and lymphangiogenesis via TAMs requires further elucidation.

Melatonin (MLT), released by the pineal gland, is a major chronobiotic hormone. Since its first introduction, multiple investigations confirmed the potent antioxidative nature of melatonin action [14]. Moreover, based on its examination in clinical trials [15], this hormone elicits minimal adverse effects. In a prior investigation, we revealed that melatonin strongly reduces inflammation and oxidative damage [16, 17]. Prior studies have suggested that melatonin exhibits significant apoptotic, angiogenic, oncostatic, and anti-proliferative effects across different types of cancer cells [18]. Therefore, we hypothesized that it would also regulate tumor-induced pyroptosis.

Herein, we examined the signaling networks and influence of melatonin on TAM-regulated tumor angiogenesis and lymphangiogenesis via inhibition of the NLRP3 axis in the lung adenocarcinoma cell TME.

## RESULTS

### *In vitro* analysis

#### THP-1 macrophage and A549 co-culture enhanced NLRP3 inflammasome axis member expression in A549 cells

**Figure 1:**
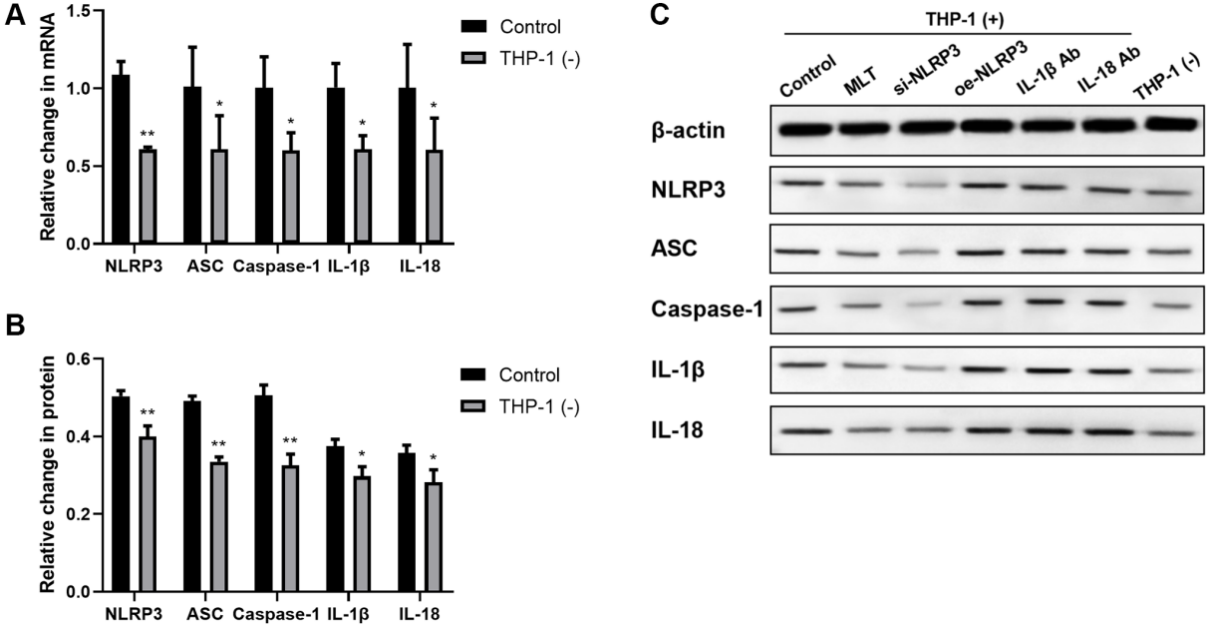
**Co-culturing THP-1 macrophages with A549 strongly enhanced the NLRP3 inflammasomal axis expressions in A549.** (**A**) The NLRP3, ASC, Caspase-1, IL-1β, and IL-18 transcript contents were substantially augmented in the A549 and THP-1 co-culture system. (**B**, **C**) The NLRP3, ASC, Caspase-1, IL-1β, and IL-18 protein expressions were upregulated in the A549 and THP-1 co-culture system, as evidenced by western blot assay. β-actin served as the endogenous control. ^*^p < 0.05, ^**^p < 0.01. Data provided as mean ± SD (*n* = 3).

To better elucidate the influence of A549 and macrophage co-culture on the NLRP3 axis, we evaluated the mRNA and protein contents of NLRP3 axis members in A549. In short, we cultured A549 cells, with or without THP-1 macrophages, for a period of 24 hours. We revealed that the NLRP3 inflammasome axis-related transcripts, such as, NLRP3, ASC, and Caspase-1, were strongly enhanced in the A549 and THP-1 co-culture system, compared to controls. We further validated these findings by examining the corresponding protein levels, using western blot analysis (Figure 1A–1C). Since the NLRP3 inflammasome is primarily involved in IL-1β and IL-18 maturation, we also assessed the IL-1β and IL-18 transcript and protein expressions in A549. Relative to control, both IL-1β and IL-18 transcript and protein expressions were strongly enhanced in the A549/THP-1 co-culture system (Figure 1A–1C).

#### Co-culturing THP-1 macrophages with A549 accelerated angiogenesis and lymphangiogenesis

**Figure 2:**
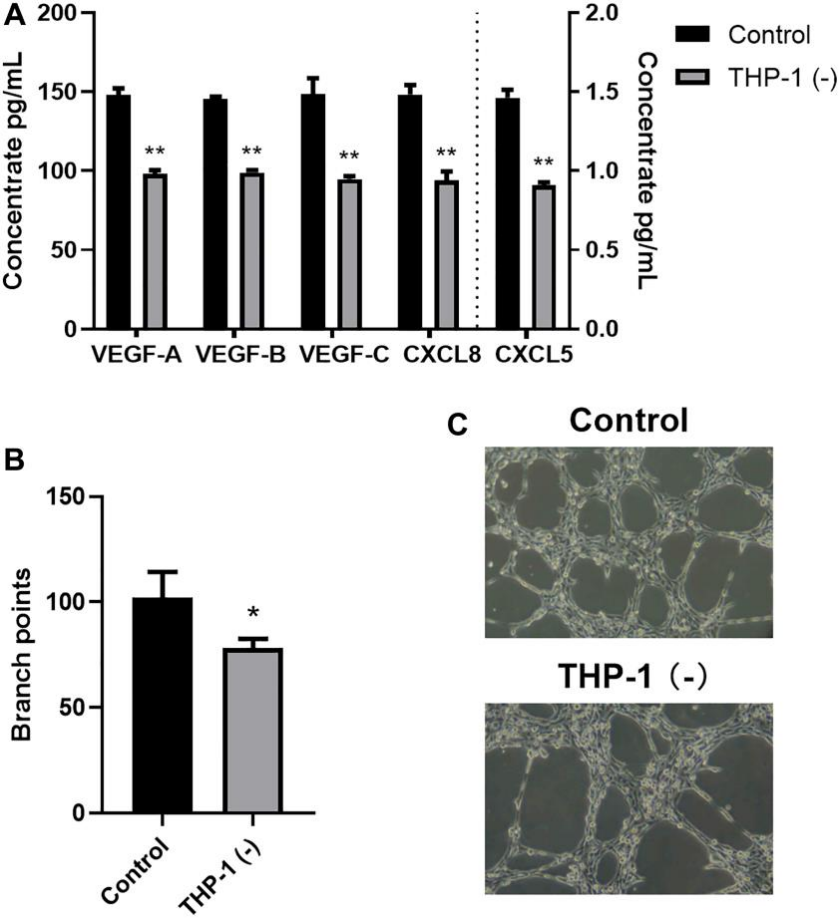
**Co-culturing THP-1 macrophages with A549 promoted TME angiogenesis and lymphangiogenesis.** (**A**) The VEGF-A, VEGF-B, VEGF-C, CXCL8, CXCL5 protein expressions, as detected by ELISA. (**B**, **C**) Branch point quantification. Typical phase-contrast images revealing tube formation. ^*^p < 0.05, ^**^p < 0.01. Data provided as mean ± SD (*n* = 3).

To explore the THP-1 macrophage-mediated regulation of tumor neovascularization and lymphangiogenesis, we next examined the VEGF and CXCL chemokine protein expressions. Following a 24-h A549 co-culture with/without THP-1 macrophage, we assessed the VEGF-A, VEGF-B, VEGF-C, CXCL5, and CXCL8 contents in the supernatant using ELISA. Relative to the A549 cells, the VEGF-A, VEGF-B, VEGF-C, CXCL5, and CXCL8 contents were markedly enhanced in the A549/THP-1 co-culture system (Figure 2A). Subsequently, we assessed the tube forming ability of LECs to examine whether THP-1 macrophages directly act on human LECs to promote lymphangiogenesis. We revealed that tube formation was strongly upregulated in the A549/THP-1 co-culture supernatants, relative to the untreated A549 culture supernatants (Figure 2B, 2C).

#### The NLRP3 inflammasome axis directly modulated tumor angiogenesis and lymphangiogenesis

**Figure 3:**
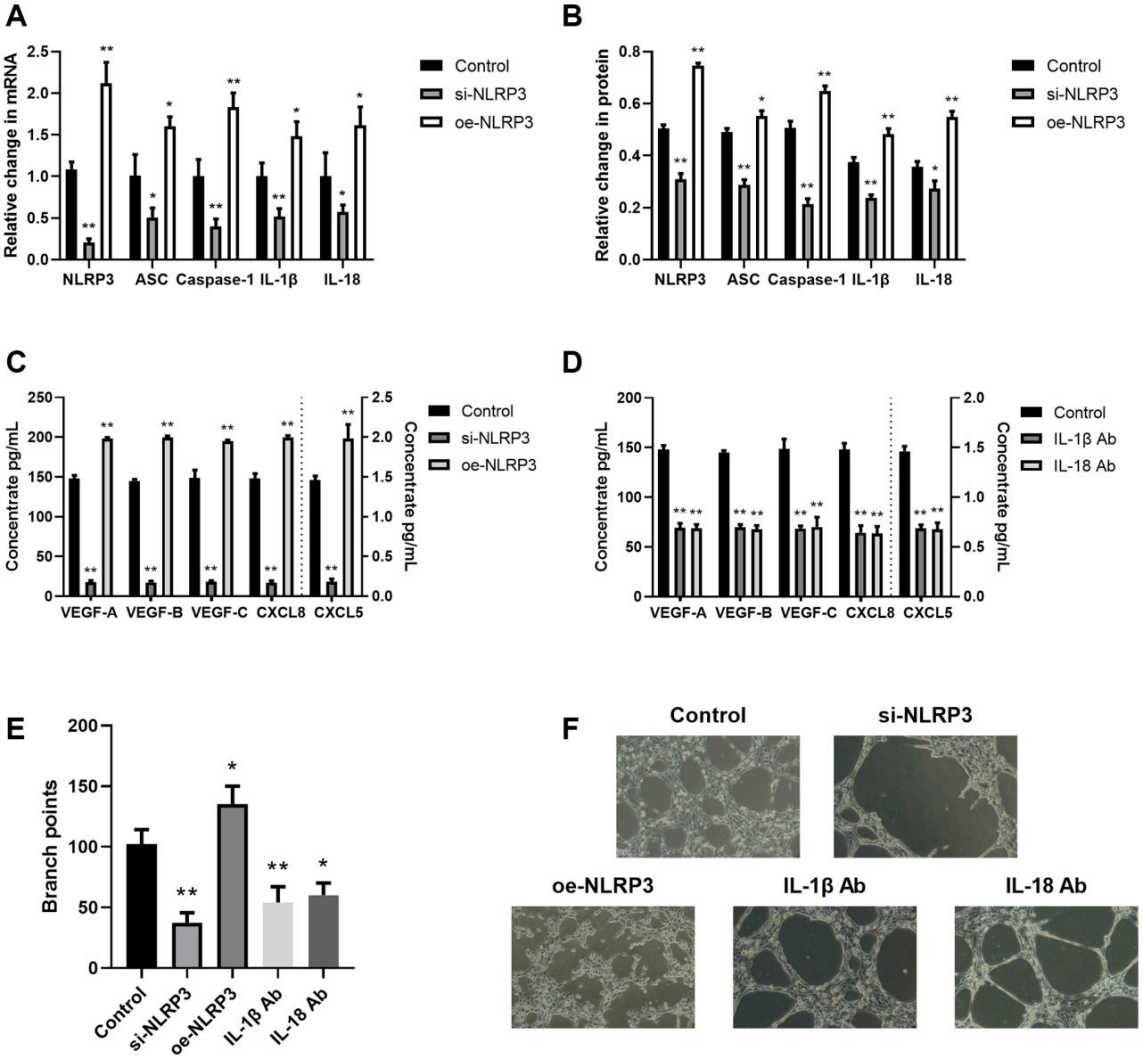
**The NLRP3 inflammasomal axis positively modulated angiogenesis and lymphangiogenesis.** (**A**) The NLRP3, ASC, Caspase-1, IL-1β, and IL-18 transcript expressions in the control, si-NLRP3, and oe-NLRP3 cells, as evidenced by RT-PCR. (**B**) The NLRP3, ASC, Caspase-1, IL-1β, and IL-18 protein contents in the control, si-NLRP3, and oe-NLRP3 cells, as evidenced by western blot assay. (**C**) The VEGF-A, VEGF-B, VEGF-C, CXCL8, and CXCL5 protein expressions in the control, si-NLRP3, and oe-NLRP3 cells, as detected by ELISA. (**D**) The VEGF-A, VEGF-B, VEGF-C, CXCL8, and CXCL5 protein contents, as detected by ELISA in control, IL-1β Ab-, and IL-18 Ab-treated cells. (**E**, **F**) Branch point quantification. Typical phase-contrast images revealing tube formation. ^*^p < 0.05, ^**^p < 0.01. Data provided as mean ± SD (*n* = 3).

Inflammation is a strict angiogenesis and lymphangiogenesis regulator [19]. Hence, we next explored the association between the NLRP3 axis and angiogenesis/lymphangiogenesis. Using NLRP3 overexpression or si-NLRP3 vector in A549 cells, we revealed that NLRP3 transcript and protein expressions, as well as downstream inflammatory cytokine (IL-1β and IL-18) expressions were significantly enhanced (oe-NLRP3) or decreased (si-NLRP3), as expected (Figure 3A, 3B). Additionally, we revealed that si-NLRP3 markedly diminished VEGF and CXCL chemokine contents, as well as LECs tube formation, whereas, oe-NLRP3 enhanced those effects (Figure 3C–3F). To elucidate whether the NLRP3 downstream signaling contributes to angiogenesis and lymphangiogenesis, we next treated the A549/THP-1 co-culture system with IL-1β Ab and IL-18 Ab. Based on our results, both IL-1β Ab and IL-18 Ab strongly suppressed VEGF and CXCL chemokine expressions, as well as LECs tube formation (Figure 3D–3F). This evidence suggested that NLRP3 stimulated TME-related angiogenesis and lymphangiogenesis using the inflammasomal axis and downstream cytokines (IL-1β and IL-18).

#### Melatonin suppressed angiogenesis and lymphangiogenesis via the NLRP3 inflammasomal axis

**Figure 4:**
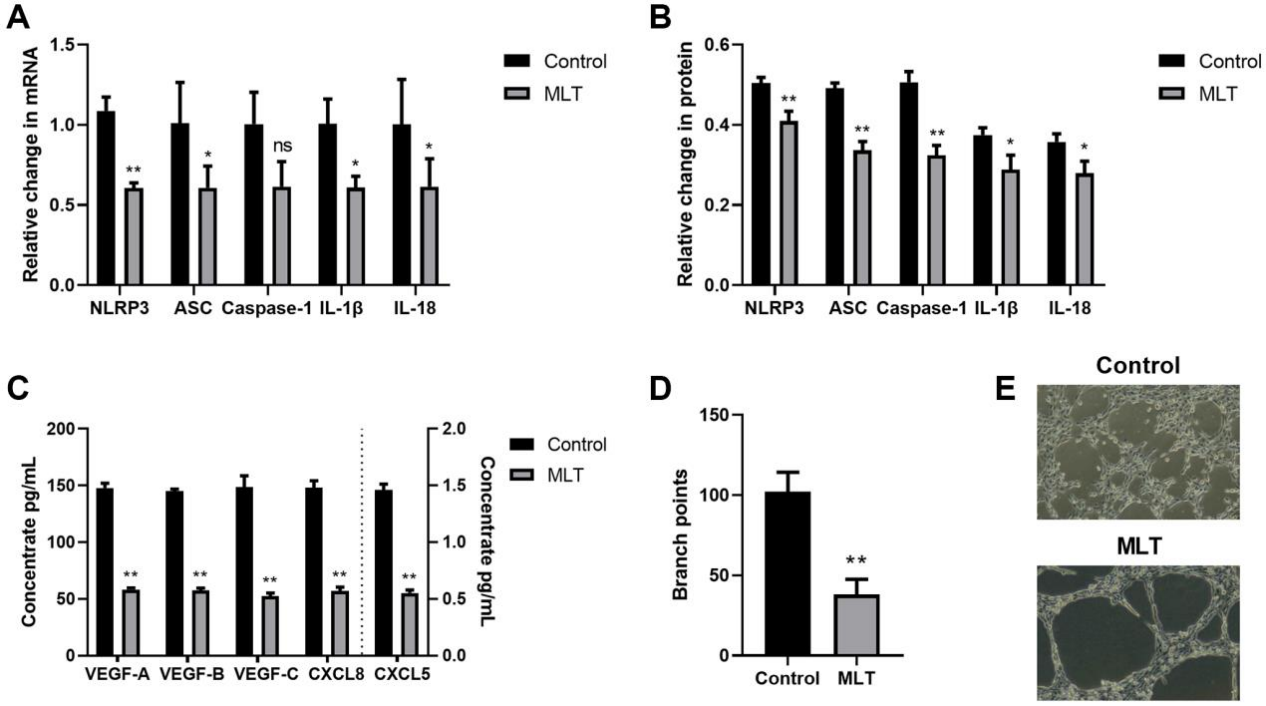
**Melatonin strongly inhibited angiogenesis and lymphangiogenesis via the NLRP3 inflammasomal axis.** (**A**) The NLRP3, ASC, Caspase-1, IL-1β, and IL-18 transcript expressions in the melatonin-treated and control cells, as evidenced by RT-PCR. (**B**) The NLRP3, ASC, Caspase-1, IL-1β, and IL-18 protein expressions in the melatonin-treated and control cells, as evidenced by western blot assay. (**C**) The VEGF-A, VEGF-B, VEGF-C, CXCL8, and CXCL5 protein contents in the melatonin-treated and control cells, as evidenced by ELISA. (**D**, **E**) Branch point quantification. Typical phase-contrast images revealing tube formation. ^*^p < 0.05, ^**^p < 0.01. Data provided as mean ± SD (*n* = 3).

In a prior investigation, we revealed that melatonin strongly inhibited inflammatory responses via its suppression of the NLRP3 inflammasomal axis [17]. Therefore, herein, we investigated the melatonin-mediated regulation of the NLRP3-modulated angiogenesis and lymphangiogenesis. To this end, we co-cultured A549 and THP-1 for 24 h with melatonin (100 μmol/L), and examined the expression levels of the NLRP3 axis. Relative to controls, melatonin strongly prevented NLRP3 inflammasomal axis mRNA and protein expressions (Figure 4A, 4B). We further assessed the melatonin-mediated regulation of angiogenesis and lymphangiogenesis. Based on our VEGF and CXCL ELISA data, melatonin induced a substantial decrease in the aforementioned proteins (Figure 4C). Furthermore, in the co-culture system, melatonin strongly inhibited LEC tube formation, relative to controls (Figure 4D, 4E). Together, these data suggested that melatonin suppressed TME angiogenesis and lymphangiogenesis, at least partly, via inhibition of the NLRP3 inflammasomal axis.

### *In vivo* analysis

#### Melatonin and NLRP3 pathway inhibited tumor growth in mice

**Figure 5:**
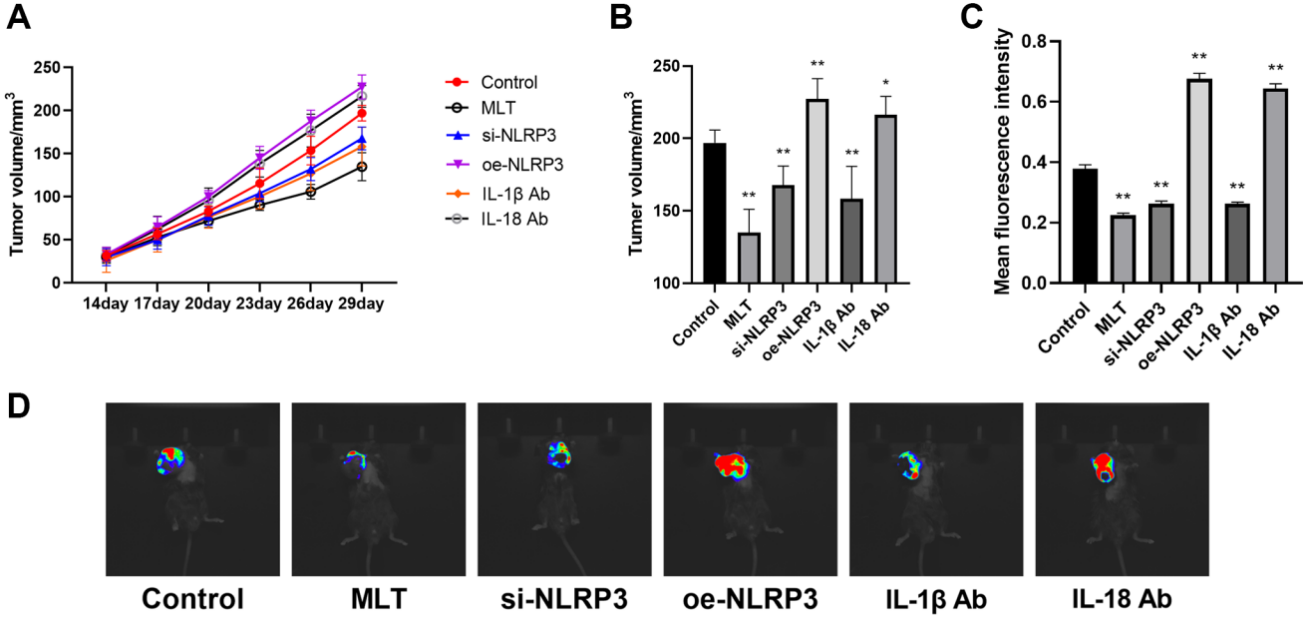
**Effects of melatonin and NLRP3 pathway modulation on tumor growth in mice.** (**A**) Subcutaneous tumor volume in mice monitored over time in different treatment groups. (**B**) Tumor volume after 30 days among different groups compared to the control group. (**C**, **D**) *In vivo* fluorescence imaging and quantification of mean fluorescence intensity in subcutaneous tumors from different groups. ^*^p < 0.05, ^**^p < 0.01, vs. control group. Data provided as mean ± SD (*n* = 3).

Further, we established a subcutaneous tumor model of Lewis Lung Carcinoma (LLC) cells in mice. In order to investigate the influence of melatonin on the NLRP3 pathway, we established the melatonin group (MLT), the NLRP3 overexpression group (oe-NLRP3), the NLRP3 knockdown group (si-NLRP3), the IL-1β antibody group (IL-1β Ab), and the IL-18 antibody group (IL18-Ab). We monitored the growth of subcutaneous tumors in mice. The study revealed that with increasing incubation time, significant differences in the volume of subcutaneous tumors were observed among the various groups compared to the control group (Figure 5A). Measurement of tumor volume after 30 days showed that compared to the control group, the subcutaneous tumor volume significantly decreased in the MLT group, si-NLRP3 group, and IL-1β Ab group, while it significantly increased in the oe-NLRP3 group and IL-18 Ab group (Figure 5B). *In vivo* fluorescence imaging also indicated that the average fluorescence intensity significantly decreased in the MLT group, si-NLRP3 group, and IL-1β Ab group, whereas it significantly increased in the oe-NLRP3 group and IL-18 Ab group (Figure 5C, 5D). The results of the IL-18 antibody group may be due to the dual role of IL-18 in tumor growth in the *in vivo* model, and its potential regulation by other pathways. Therefore, in subsequent experiments, we did not include the IL-18 ab group.

#### Melatonin inhibited the expression of the NLRP3 pathway in mice

**Figure 6:**
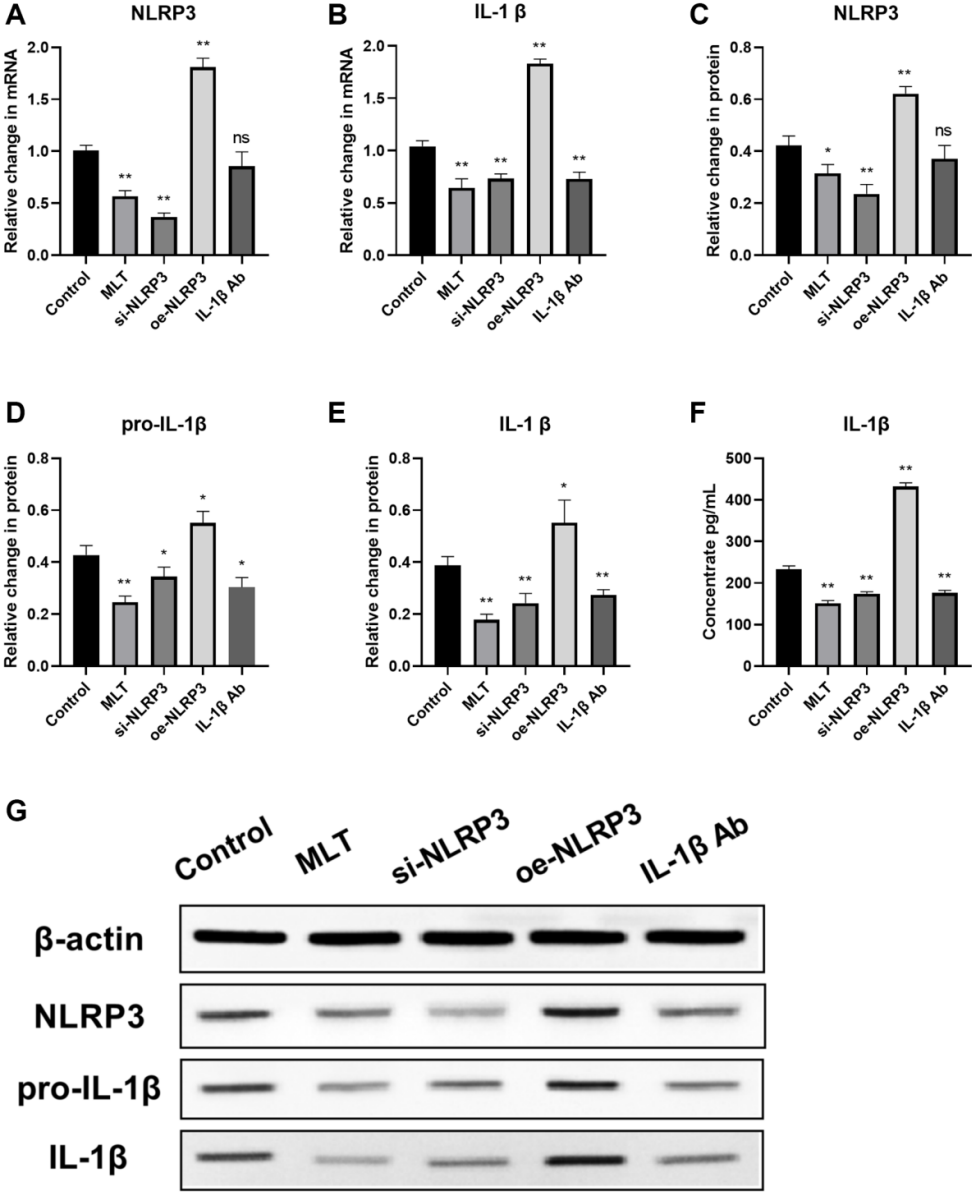
**Melatonin inhibited the expression of the NLRP3 pathway in tumor tissues and peripheral blood of mice.** (**A**, **B**) mRNA expression levels of NLRP3 pathway components (NLRP3 and IL-1β) in tumor tissues evaluated by RT-PCR. (**C**–**F**) Protein expression levels of NLRP3 pathway components (NLRP3, pro-L-1β and IL-1β) in tumor tissues assessed by western blotting. (**G**) Expression levels of IL-1β in peripheral blood of mice analyzed by ELISA. ^*^p < 0.05, ^**^p < 0.01, vs. control group. Data provided as mean ± SD (*n* = 3).

Then we investigated the regulatory effect of melatonin on the NLRP3 pathway *in vivo*. After 30 days of incubation, mice were euthanized and tumor tissue specimens were harvested. The mRNA and protein levels of NLRP3 pathway in the tumor tissues of each group were evaluated using RT-PCR and western blotting, respectively. Our findings indicate that both MLT group and si-NLRP3 group significantly suppressed the mRNA and protein expression levels of NLRP3 and downstream IL-1β in tumor tissues. Conversely, the oe-NLRP3 group exhibited opposite results. In the IL-1β Ab group, the mRNA and protein expression levels of IL-1β were suppressed (Figure 6A–6F). Meanwhile, we assessed the expression of IL-1β in the peripheral blood of mice. The results showed that the expression levels of IL-1β were significantly decreased in the MLT group, si-NLRP3 group, and IL-1β Ab group, while the oe-NLRP3 group exhibited opposite results (Figure 6G).

#### Melatonin downregulated angiogenesis and lymphangiogenesis by targeting the NLRP3 pathway in mice

**Figure 7:**
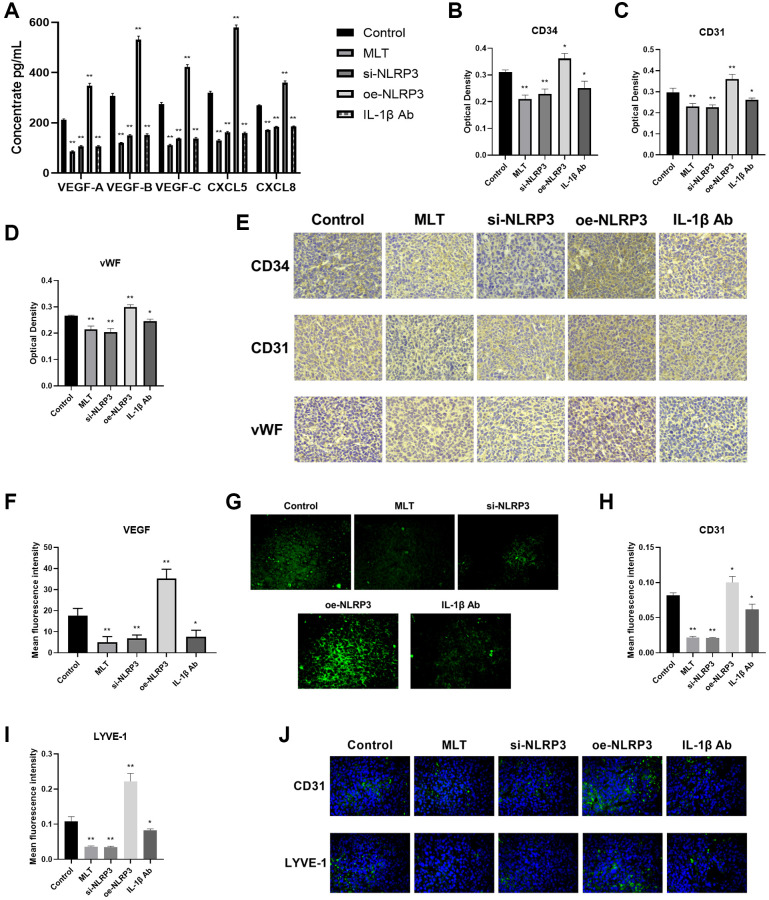
**Melatonin downregulated angiogenesis and lymphangiogenesis by targeting the NLRP3 pathway in mice.** (**A**) Levels of angiogenic and lymphangiogenic factors (VEGF-A, VEGF-B, VEGF-C, CXCL5, and CXCL8) in mice serum determined by ELISA. (**B**–**E**) Immunohistochemical (IHC) staining of vascular marker proteins (CD34, CD31, and vWF) in tumor tissues. (**F**, **G**) Immunofluorescence (IF) detection of VEGF in tumor tissues. (**H**–**J**) IF detection of neovascularization marker (CD31) and lymphangiogenesis marker (LYVE-1) in tumor tissues. ^*^p < 0.05, ^**^p < 0.01, vs. control group. Data provided as mean ± SD (*n* = 3).

Then, we further investigated the effects of melatonin and the NLRP3 pathway on tumor angiogenesis and lymphangiogenesis. Initially, we evaluated the levels of VEGF-A, VEGF-B, VEGF-C, CXCL5, and CXCL8 in mice serum using ELISA. The study revealed that compared to the control group, the expression levels of these proteins were significantly decreased in the MLT group, si-NLRP3 group, and IL-1β Ab group, while exhibiting opposite trends in the oe-NLRP3 group (Figure 7A). CD34, CD31, and vWF are commonly used vascular marker proteins, and immunohistochemical (IHC) staining of these proteins can detect vascular formation in tumor tissues. Our research found that compared to the control group, the optical density of CD34, CD31, and vWF were significantly reduced in the MLT group, si-NLRP3 group, and IL-1β Ab group, while showing opposite trends in the oe-NLRP3 group (Figure 7B–7E). Furthermore, we conducted immunofluorescence (IF) detection of VEGF in tumor tissues. The findings indicated that the average fluorescence intensity of VEGF was significantly decreased in the MLT group, si-NLRP3 group, and IL-1β Ab group, while showing opposite results in the oe-NLRP3 group (Figure 7F, 7G). CD31 and LYVE-1 serve as markers for neovascularization and lymphangiogenesis, respectively. IF detection of tumor tissues was also performed for these markers. Consistent with previous results, we observed a significant decrease in the average fluorescence intensity of CD31 and LYVE-1 in the MLT group, si-NLRP3 group, and IL-1β Ab group, while demonstrating opposite trends in the oe-NLRP3 group (Figure 7H–7J).

## DISCUSSION

Angiogenesis and lymphangiogenesis serve essential functions in tumorigenesis and LC progression [2, 3]. Angiogenesis represents new blood vessel formation from pre-existing vessels. Lymphangiogenesis is critical for lymphatic vessel system development, and it is a major contributor to lymph node metastasis. Prior investigations revealed that TAM invasion of the TME is strongly correlated with angiogenesis and lymphangiogenesis [20, 21]. However, the underlying mechanisms remain undetermined. Herein, we demonstrated that the NLRP3 axis expression was markedly enhanced in A549 cells once it was co-cultured with THP-1 macrophages. NLRP3 upregulation also strongly correlated angiogenesis and lymphangiogenesis acceleration, and this was considerably inhibited by melatonin. The experimental outcomes also underwent verification in animal models to ensure the reliability of our conclusions.

TAMs are crucial to the tumor-associated inflammation, and they are prevalent among inflammatory cells within the TME [6]. They establish a crucial crosstalk between tumor and nearby stromal cells. The NLRP3 inflammasome is a complex of proteins belonging to the innate immune system, and it is absolutely necessary for IL-1β and IL-18 maturation [9]. Prior investigation revealed that the NLRP3 inflammasomal suppression using genetic deficiency or pharmacological inhibitor substantially suppresses the metastatic potential of tumor cells [22]. Several reports indicated that the TAM-associated NLRP3 activation enhances both tumor development and progression [12, 23]. Based on our *in vitro* investigation, NLRP3 inflammasomes (NLRP3, ASC and Caspase-1) and its downstream inflammatory factors (IL-1β and IL-18) expressions were markedly enhanced in the A549- THP-1 co-culture, as opposed to controls. Thus, we further examined the role of A549/THP-1 co-cultivation on angiogenesis and lymphangiogenesis.

TAM invasion is intricately linked to the vascular density, recurrence rate, and overall survival of several malignant tumors [24, 25]. In breast cancer and pancreatic tumor models, Mazzieri et al. demonstrated that the ANG2-TIE2 network promotes cell-to-cell crosstalk between TIE2-expressing macrophages and endothelial cells, and this crosstalk strongly modulated tumor angiogenesis and development [26]. Lin et al. reported that CCL18, secreted from TAMs, synergistically enhanced endothelial cell migration and angiogenesis with VEGF, which, in turn, accelerated tumor progression [27]. Based on our findings, the A549 and THP-1 macrophage co-culture strongly stimulated VEGF-A, VEGF-B, CXCL5, and CXCL8 expressions. Moreover, the upregulated cytokine expressions were intricately related to the TME neovascularization and angiogenesis [28, 29]. Tumor-related lymphangiogenesis is also involved in tumor growth, neovascularization, tumor invasion, and lymph node metastasis [30, 31]. In an investigation involving NSCLC by Hwang et al., TAMs invasion was related to peritumoral lymphangiogenesis and poor prognosis [2]. Herein, we demonstrated that the THP-1/A549 co-culture system strongly upregulated VEGF-C expression and LECs tube formation. Emerging evidence also indicated that VEGF-C strongly modulates lymphangiogenesis and stimulates lymphatic endothelium proliferation [32]. Fischer et al. revealed that the anti-PlGF antibody essentially blocks macrophage recruitment to orthotopic pancreatic tumors, reduces TAMs recruitment, as well as tumor VEGF-C levels, which, in turn, diminishes both lymphatic vessel density and lymph node metastasis [33]. Herein, we also confirmed that an A549 and THP-1 co-culture strongly enhanced TME angiogenesis and lymphangiogenesis. However, additional investigations are necessary to elucidate the specific underlying mechanisms.

Inflammation has a close relationship with tumor angiogenesis and lymphangiogenesis. Inflammation recruits inflammatory cells to the TME, and releases multiple pro-inflammatory mediators that modulate endothelial cell growth and migration, thereby enhancing tumor angiogenesis and lymphangiogenesis [34, 35]. Chai et al. reported that NLRP3 over-expression induced a marked rise in pro-inflammatory cytokine and VEGF expressions [36]. Weichand et al. revealed that the S1PR1 axis in macrophages augmented lymphangiogenesis via NLRP3/IL-1β in breast tumors [37]. Given these evidences, herein, we employed the si-NLRP3/oe-NLRP3/IL-1β Ab/IL-18 Ab to examine the NLRP3 axis significance in TME angiogenesis and lymphangiogenesis using the co-culture system. We revealed that the NLRP3 inflammasomal axis strongly enhanced tumor-related angiogenesis and lymphangiogenesis in the A549/THP-1 co-culture system. In addition, in a subcutaneous tumor model of lung cancer in mice, we observed that NLRP3 inflammasome and downstream IL-1β could promote tumor angiogenesis and lymphangiogenesis, as well as tumor growth.

More recently, overwhelming research suggested a strong anti-cancer role of melatonin. The melatonin-mediated anti-cancer actions are typically correlated with mechanisms, such as, antioxidant activity, apoptotic modulation, tumor metabolism, tumor immunity, as well as angiogenic and migratory inhibition [38]. Prior investigations revealed that melatonin enhanced LC cell apoptosis via inhibition of the HDAC1 axis, augmentation of caspase-3 activity, and suppression of Bcl-2 and GSH expressions [39]. Chao et al. revealed that melatonin regulates PD-L1 expression and impacts tumor immunity in KRAS-mutant non-small cell lung cancer. [40]. In addition, using human lung adenocarcinoma cell line, Wang et al. revealed that melatonin reduces circ_0017109 expression and inhibits migration, invasion, and proliferation of non-small cell lung cancer cells by downregulating TOX3 through direct activation of miR-135b-3p [41]. Unfortunately, till date, there is limited literature on the anti-angiogenic activities of melatonin in NSCLC. Our previous report suggested that melatonin suppresses NLRP3 inflammasomal activation via oxidative stress inhibition [17]. Hence, herein, we speculated that melatonin potentially alleviates tumor-associated angiogenesis and lymphangiogenesis using the NLRP3 axis. Our mechanistic analyses revealed that melatonin indeed suppressed the NLRP3 axis, decreased VEGF and CXCL cytokine contents, and inhibited LECs tube formation in the A549/THP-1 co-culture system. *In vivo* study, we discovered that melatonin can suppress tumor angiogenesis and lymphangiogenesis, as well as tumor growth, by downregulating the expression of the NLRP3 pathway. Given this evidence, a melatonin-mediated targeting of the NLRP3 axis may be a promising approach to preventing TAM-regulated angiogenesis and lymphangiogenesis in lung adenocarcinoma.

In conclusion, a THP-1 and A549 cell co-culture strongly enhanced angiogenesis and lymphangiogenesis. Therefore, targeted modulation of the NLRP3 axis is a potential strategy for TAMs-associated angiogenesis and lymphangiogenesis suppression. Herein, we proposed a model whereby melatonin protected against angiogenesis and lymphangiogenesis via the NLRP3 axis in a A549/THP-1 co-culture system. In a subcutaneous tumor model in mice, melatonin has also been observed to elicit marked inhibitory effects on tumor angiogenesis, lymphangiogenesis, and tumor growth through modulation of the NLRP3 axis. Melatonin administration may therefore be a potential promising therapeutic regimen for lung adenocarcinoma patients.

## METHODS

### Cell lines and culture

A549, THP-1 and primary human lymphatic endothelial cells (LECs) were acquired from Procell Life Science and Technology Co., Ltd. (Hubei, China). A549 cells were incubated in Dulbecco’s modified Eagle’s medium, containing 10% fetal bovine serum (FBS; Procell Life Science and Technology Co., Ltd., Hubei, China). THP-1 was grown in RPMI-1640 medium containing 10% FBS. LECs were incubated in Endothelial Cell Medium with 10% FBS, and 1% endothelial cell growth factors. All media were sourced from Procell Life Science and Technology Co., Ltd., Hubei, China, and all cells were placed in a 37°C humid chamber with 5% CO_2_ and 95% air. A549 cells were incorporated with NLRP3 siRNA or overexpression vector, prior to co-culture with THP-1. Additionally, the A549/THP-1 co-culture systems were treated in advance with melatonin (100 μmol/L), as well as the IL-1β (IL-1β Ab) and IL-18 antibodies (IL-18 Ab).

### Animal studies

The animal studies adhered to the Animal Management Rules of the Chinese Ministry of Health, and were approved by the Animal Care Committee of Peking Union Medical College (XHDW-2021-066). Male C57BL/6 mice were obtained from the Experimental Animal Center of Peking Union Medical College Hospital. Mice were provided with standard rodent chow and tap water ad libitum and housed in a facility with controlled environmental conditions (25–28°C, 12-hour light/dark cycle).

LLC-luciferase cells (5 × 10^6^ cells/mouse) were subcutaneously injected into the right forelimb axillary region of each mouse, followed by group-specific interventions. Mice were randomly divided into six groups (*n* = 6 per group). All groups underwent subcutaneous tumorigenesis. The control group received no additional interventions. Mice in the melatonin group received melatonin (Sigma-Aldrich, MO, USA) via intraperitoneal injection (100 mg/kg/day). NLRP3 inhibition was achieved by administering NLRP3 small interfering RNA via tail vein injection. In the NLRP3 overexpression group, the LLC-luciferase cell line was stably overexpressing NLRP3 through lentivirus infection and selection with hygromycin treatment. IL-1β and IL-18 monoclonal antibody control groups received intraperitoneal injections of IL-1β monoclonal antibody and IL-18 monoclonal antibody, respectively (100 μg/kg/day). After 30 days, the animals were euthanized, and relevant specimens were collected and processed. The samples were either fixed in 4% paraformaldehyde (PFA) for 24 hours or stored in liquid nitrogen.

### Co-culture system

We utilized 6-well plates and Transwell chambers to co-culture A549 cells and THP-1 macrophages. In short, THP-1-containing Transwell chamber was placed on top of A549-containing (in RPMI-1640 and 10% FBS) 6-well plate. The co-culture was incubated for 24 h. Lastly, co-culture without THP-1 served as the control.

### Quantitative polymerase chain reaction (qPCR)

Total RNA extraction from A549 cells utilized TRIzol (Invitrogen, CA, USA), and RNA quantification was done using a NanoDrop 2000 instrument (Thermo Fisher Scientific, Bremen, Germany). cDNA synthesis utilized SuperScript III RT (Invitrogen, CA, USA). Oligo(dT), and Applied Biosystems StepOne Real-Time PCR instrument (Applied Biosystems Inc., CA, USA) conducted the qRT-PCR reactions using SYBR Mix (Invitrogen) and the following targeted primers: NLRP3, F 5′-AAGGAAGTGGACTGCGAGAA-3′ and R 5′-AACGTTCGTCCTTCCTTCCT-3′; ASC, F 5′-GTCACAAACGTTGAGTGGCT-3′ and R 5′-ACTGAAGAGCTTCCGCATCT-3′; Caspase-1, F 5′-GGCATGACAATGCTGCTACA-3′ and R 5′-TCTGGGACTTGCTCAGAGTG-3′; IL-1β, F 5′-CTCTCTCCTTTCAGGGCCAA-3′ and R 5′-GCGGTTGCTCATCAGAATGT-3′; IL-18, F 5′-TCACCAGAGGTCAGGTGTTC-3′ and R 5′-TCCGGAGTGCAAGTGATTCT-3′; β-actin, F 5′-CCAGCCTTCCTTCTTGGGTA-3′ and R 5′-CAATGCCTGGGTACATGGTG-3′. β-actin was used as an internal control.

### Western blot

RIPA buffer (MDL Biotech, Beijing, China) containing 2% SDS, 10% glycerol, 62.5 mmol/L Tris-HCl; pH 6.8 was employed for A549 cell lysis and total protein extraction. The resulting lysate was centrifuged for 15 min at 4°C and 12,000 × g, prior to supernatant collection and protein assessment with the BCA assay (MDL Biotech). 20 μg protein separation was carried out in 15% SDS-PAGE, prior to PVDF membrane transfer, which was overnight (ON) incubated at 4°C in primary antibodies targeting the following: NLRP3, IL-1β, and IL-18 (Affinity Biosciences, OH, USA), as well as ASC and Caspase-1 (Cell Signaling Technology, MA, USA), and β-actin (MDL Biotech, Beijing, China). Following 3 PBS rinses, the membrane was further incubated for 1 h in HRP-linked goat anti-mouse/anti-rabbit IgG at room temperature (RT), prior to protein visualization and analysis with enhanced chemiluminescence and ImageJ, respectively.

### ELISA

Supernatant VEGF and CXCL chemokines quantification utilized corresponding ELISA kits (USCN Business Co., Ltd., Hubei, China) and associated instructions. The levels of IL-1β, VEGF and CXCL chemokines in mouse serum were also assessed via ELISA kits (USCN Business Co., Ltd., Hubei, China).

### Tube forming assay

Matrigel tube forming assay was used to determine cellular tube forming ability. Following intervention, LECs (1 × 10^5^ cells) were plated onto Matrigel-precoated (50 μl for 45 min at 37°C) 96-well culture plates, then observed under an inverted fluorescence microscope (NIB610-FL). Branchpoint quantification utilized the Angiogenesis Analyzer using ImageJ.

### *In vivo* imaging

Subcutaneous tumors were established in mice by injecting LLC-luciferase cells. After 30 days, *in vivo* imaging was conducted following the administration of isoflurane anesthesia to the animals. The fluorescence intensity was measured using the VISQUE *in vivo* Smart-LF imaging system (Vieworks, Anyang, Korea).

### Immunohistochemistry

For IHC analysis, paraffin-embedded tissue sections were mounted on glass slides and subsequently deparaffinized. Following a 30-minute blocking step at room temperature, sections were probed overnight at 4°C with primary anti-CD34 antibody (1:50, Abcam, MA, USA, Cat. No. ab8158), anti-CD31 antibody (1:100, Abcam, MA, USA, Cat. No. ab222783) and anti-vWF antibody (1:50, Abcam, MA, USA, Cat. No. ab287962). Subsequently, sections were probed with HRP-linked secondary goat anti-rabbit IgG (1:200 dilution, MDL Biotech, Beijing, China), followed by incubation with the 3,3′-diaminobenzidine tetrachloride chromogen as a substrate at room temperature. Three randomly selected fields from each section were assessed in a blinded manner. The expression levels of the factors were quantified using integrated optical density values measured with ImageJ software (http://imagej.net/ImageJ).

### Immunofluorescence

Tumor tissue sections were deparaffinized and antigen retrieval was performed by heating the slides in citrate buffer (pH 6.0) at 95°C for 20 minutes. Nonspecific binding was blocked with blocking solution for 1 hour at 37°C. Subsequently, the sections were incubated with primary antibodies overnight at 4°C: anti-CD31 antibody (1:100, Abcam, MA, USA, Cat. No. ab222783), anti-LYVE-1 antibody (1:100, Santa Cruz, CA, USA, Cat. No. sc-65647) and anti-VEGF antibody (1:50, Santa Cruz, CA, USA, Cat. No. sc-57496). The following day, sections were washed three times with PBS and incubated with corresponding fluorochrome-conjugated secondary antibodies (1:200, Jackson Immunoresearch, PA, USA) for 1 hour at 37°C in the dark. Sections were then washed three times with PBS and counterstained with DAPI for 10 minutes in the dark. After a final wash with PBS, sections were mounted with glycerol and immediately observed under a fluorescence microscope (Nikon, ECLIPSE Ci, Tokyo, Japan).

### Statistical analysis

Data statistical evaluation utilized student’s *t*-test, and they are provided as mean ± SD. SPSS (version 19.0, SPSS software, Munich, Germany) was used. *p* < 0.05 was adjusted as significance threshold.
